# Activities and Effects of Ergot Alkaloids on Livestock Physiology and Production

**DOI:** 10.3390/toxins7082801

**Published:** 2015-07-27

**Authors:** James L. Klotz

**Affiliations:** Forage-Animal Production Research Unit, Agricultural Research Service, United States Department of Agriculture, Lexington, KY 40546, USA; E-Mail: james.klotz@ars.usda.gov; Tel.: +1-859-257-1647; Fax: +1-859-257-3334

**Keywords:** ergot alkaloids, livestock, physiology, production

## Abstract

Consumption of feedstuffs contaminated with ergot alkaloids has a broad impact on many different physiological mechanisms that alters the homeostasis of livestock. This change in homeostasis causes an increased sensitivity in livestock to perturbations in the ambient environment, resulting in an increased sensitivity to such stressors. This ultimately results in large financial losses in the form of production losses to livestock producers around the world. This review will focus on the underlying physiological mechanisms that are affected by ergot alkaloids that lead to decreases in livestock production.

## 1. Introduction

As secondary metabolites of the *Claviceps* and *Epichloë* spp. of fungi, the ergot alkaloid mycotoxins have a significant impact on livestock health and productivity around the world. The geographic scope of this impact ranges from countries such as the United States [1], New Zealand [2], and Australia [3] that have a heavy reliance on grazing for aspects of livestock production. This impact also extends to countries such as Japan [4,5], Korea [6], and the United Arab Emirates [7] that import hay as a feedstuff for livestock production and maintenance and countries such as Australia that use ergot-contaminated feeds in intensive livestock production settings [8,9]. A substantial challenge in the elucidation of ergot alkaloid-induced effects is the highly variable individual animal response to exposure. This variation is in large part due to the complex plant–fungus–animal–microbe–environment interaction that results in changing alkaloid concentrations, alkaloid proportions, availability, and distribution of various isomeric forms throughout the animal [10,11,12,13,14]. From the livestock side of the interaction, this can cause issues that range from the often-unpredictable acute outbreaks of gangrenous ergotism (*i.e.*, fescue foot [15] that results in loss of extremities such as hooves, ear tips, and tail switches), to more subtle and chronic decreases in livestock productivity (*i.e.*, summer slump [16], which is characterized by decreases in intake, liveweight gain, circulating prolactin, reproductive performance, milk production, and hyperthermia), and fat necrosis [17], which is often only diagnosed following necropsy. Beyond the range of symptomatic forms that ergot alkaloids manifest, there is also diversity in the manner in which symptoms are presented across animals within the same group. This suggests there exists a potential interaction between an individual’s genetic predisposition, prior exposure to ergot alkaloids, or prior health issues such as damage to hepatic or respiratory tissues that may reduce alkaloid tolerance in an individual. Additionally, aspects of the ambient environment such as temperature and humidity can influence a herd’s susceptibility to ergot toxicosis. This review will collectively discuss the ergotism and fescue toxicoses caused by the *Claviceps*-derived ergotamine and ergocristine and *Epichloë*-derived ergovaline, respectively, and focusing primarily on the animal aspects and activities associated with these ergot alkaloids. Specifically, how they disrupt normal biological processes, and result in the observed symptoms associated with ergot toxicosis in livestock.

## 2. Ergot Alkaloid Activity

**Figure 1:**
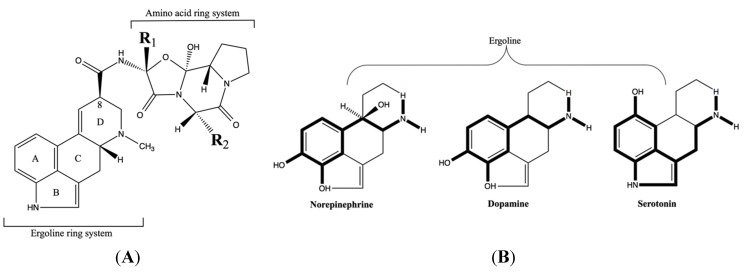
(**A**) The tetracyclic ergoline ring common to all ergot alkaloids that is variously substituted on the C-8 which in this case has an amino acid ring system that varies at the R_1_ and R_2_ substituents to create the various ergopeptine alkaloids. (**B**) The structural similarities between the ergoline ring and the catecholamines norepinephrine, dopamine, and serotonin (in bold). Reproduced from Berde and Stürmer [22]. Copyright 1978, Springer-Verlag.

Variations in ergot alkaloid type and concentration in the live plant [18,19] and in harvested plants [20] that are available for consumption will contribute to a varied alkaloid dose, but not necessarily account for the multiplicity of effects that have been generally attributed to ergotism in livestock. Rather, this diversity of effects is due to an interruption of a number of different biological processes by ergot alkaloids. The ability of ergot alkaloids to accomplish this “interruption” is directly related to the structural similarities of the tetracyclic ergoline ring (Figure 1A) common to naturally occurring ergot alkaloids [21] and similar to the ring structure of the biogenic amine neurotransmitters norepinephrine, dopamine, and serotonin (Figure 1B) [22,23]. The receptors associated with these biogenic amines are G protein-coupled membrane proteins with seven transmembrane helices [24] that consist of numerous families and subtypes. For example, serotonin receptors consist of 14 subtypes that are divided into seven families [25] that are located throughout the body in populations that vary across tissue type and this adds to the complexity of how ergot alkaloids elicit an effect.

The receptor heterogeneity of ergot alkaloids has made describing the pharmacologic profile complex. Further, as ligands, ergot alkaloids can interfere at more than one receptor site [22] and the basic activity will vary from alkaloid to alkaloid [26,27] as a result of slight structural differences [28]. As ligands, ergot alkaloids can also act as agonists (stimulatory), partial agonists, and antagonists at the same receptor sites [29]. Consequently, there are a large number of outcomes in terms of ergot alkaloid-receptor interactions. It is these receptor–alkaloid interactions that result in the diversity of problems attributed to ergot alkaloids But, what happens when an ergot alkaloid binds to one of these receptors? How does an ergot alkaloid bound to a biogenic amine receptor lead to an interruption of neurotransmission that results in a larger interruption of a biological process?

If an alkaloid like ergovaline binds to a receptor in vascular smooth muscle, it will initially act in a full agonistic manner reaching the maximal stimulation possible [30], whereas structurally similar compounds like ergocristine and ergocornine act more like partial agonists producing about 40% to 50% stimulation in the same vascular system [27]. There is also a group of reports that indicates that ergopeptine alkaloids develop a long-lasting (>2 to 3 h) vascular response [30,31,32,33]. This long-lasting vascular response appears to overlap with other data that demonstrate ergot alkaloids can act in an antagonistic manner and block serotonin-induced vascular responses in bovine basilar arteries [22], the bovine mesenteric artery and vein [34], and the rat tail artery [33]. If the ergot alkaloid–receptor complex persists, the non-competitive antagonism created by the presence of ergot alkaloids such as ergovaline will prevent the receptor from performing its designed function. This will result in receptor desensitization that has been shown to be caused by prolonged stimulation of serotonin receptors and triggers a reduced signal transduction [35]. Furthermore, the eventual receptor internalization will occur as biogenic amine receptors like serotonin (5HT) receptor 5HT_2A_ have been shown to have a rate of internalization that is accelerated by agonist occupancy [36]. This sustained binding also creates an opportunity for the accumulation of ergot alkaloids in a tissue bed. If the rate of ergot alkaloid accumulation on receptors exceeds the rate of receptor recycling or turnover, then larger impacts on biological processes can occur. Thus, continuous consumption of ergot alkaloids could result in a chronic exposure that if high enough could cause the receptor accumulation of ergot alkaloids.

The interruption of biological processes appears to extend beyond the aforementioned interaction with biogenic amine receptors. The capacity for glutamatergic neurotransmission is reduced in bovine synaptic vesicles exposed to ergovaline [37]. Also, cattle exposed to ergot alkaloids (via tall fescue consumption) appear to have impaired nucleoside metabolism as the alkaloids bromocriptine and ergovaline have been shown to inhibit bovine nucleoside transporters [38]. Ergot alkaloids have a demonstrated apoptotic effects on human primary cells in culture with ergocristine producing the strongest toxic potential out of those tested [39]. In addition to an uptake of ergotamine/ergotaminine and ergocristine/ergocristinine into the human primary cells, a separate study revealed that these ergopeptine alkaloids are capable of crossing the blood–brain barrier only in the 8-(*R*) form [40]. Under various conditions, ergot alkaloids can isomerize at the C-8 position (Figure 1a) [41]. Conversely, the epimer ergocristinine (8-(*S*) form) was unable to cross the cell barrier and significant levels of cellular accumulation and a negative influence on barrier integrity were reported [40,42]. This is a significant finding, as the 8-(*S*) forms of ergopeptine alkaloids were previously thought of as inactive [22,41]. Thus, symptoms of ergot alkaloid exposure demonstrated by an animal will depend on the type and location of the receptor, the quantity of alkaloid bound, the structural conformation of those ergot alkaloids and how this coincides with other environmental forces or stressors acting upon the animal.

## 3. Gangrenous Ergotism

Gangrenous ergotism (*i.e.*, fescue foot or fescue lameness in livestock) is one of the most acute and obvious visible effects of ergot alkaloid exposure and is a result of general blood vessel vasoconstriction and dysfunction (reviewed by Strickland *et al.* [43]). Historically, this aspect of ergotism in humans has been referred to as St. Anthony’s fire. This form of ergot poisoning is reported to consist of intense burning pain and cyanosis of the extremities [44] and is a likely reason that affected livestock are often found standing in ponds or mud wallows. This malady consists of tissue necrosis or dry gangrene of the ear tips and tail that can result in loss of the tail switch [15] and, in extreme cases, loss of affected hooves (Table 1). Advanced cases of hoof-loss commonly result in a total loss of the animal.

**Table 1:** A review of ergot alkaloid levels associated with gangrenous ergotism or fescue foot.

Alkaloid	Intake/dose (mg/kg body weight)	Animal (affected/exposed)	Effect	Source
Ethanolic extract ^1^	Not determined	Cattle (5/10)	Lameness, swelling and discoloration of coronary bands, discoloration of tail tip	[45]
Ergovaline	0.016 ^2^	Steer (1/2)	Visible inflammation of coronary band and elongation of rear hooves	[46]
Ergovaline	0.011 ^2^	Ewe (1/14)	Lameness and visible inflammation around coronary bands	[46]
Ergovaline	0.009 ^3^	Cow (1/60)	Developed lameness diagnosed as fescue foot after 55 d on treatment, complete recovery after removal from study	[47]
Ergotoxine ^4^	25.0 ^5^	Rat (20%)	Tail gangrene was observed 5 to 7 d following a single i.p. dose	[48]
Ergotamine	1.0 ^6^	Sheep (4/6)	Four out 6 died after 10d; tongue necrosis and hemorrhages around fetlock and metatarsal regions	[49]

^1^ An early study that thoroughly described the gangrenous ergotism associated with tall fescue occurred prior to the realization that ergot alkaloids were the responsible agents. The extract was made from tall fescue hay and administered as an intraperitoneal infusion; ^2^ Dose of ergovaline was estimated based on mean BW and an assumed dry matter intake level of 2% of BW and the dietary concentrations 825 ppb for steers and 540 ppb ergovaline for the ewes reported by Tor-Agbidye *et al.* [46]; ^3^ Dose of ergovaline was estimated for this cow based on mean BW and an assumed dry matter intake level of 2% of BW and the dietary concentration of 449 ppb ergovaline reported by Merrill *et al.* [47]; ^4^ Ergotoxine is a mixture of ergocornine, ergocryptine, and ergocristine; ^5^ Rats were given a single intraperitoneal (i.p.) injection of ergotoxine; ^6^ Daily total of an oral dose of ergotamine tartrate that was subdivided and administered as 3 three smaller doses.

### 3.1. Blood Flow

These clinical manifestations are a direct consequence of the largely adrenergic blockade caused by alkaloids like ergotamine (e.g., *Claviceps purpurea*) and ergovaline (e.g., *Epichloë coenophiala*) that result in a persistent vasoconstriction. This is caused by a general decrease in blood flow to the extremities and can result in damage to the vessel’s endothelial lining, edema, and thrombosis [44]. Interestingly, horses do not seem to suffer from this aspect of ergot alkaloid exposure [50], although blood flow in the distal palmar artery has been reduced in horses receiving ergot alkaloids in their diet [51]. In sheep and cattle, however, this will result in the swelling of the fetlock (the joint between the cannon bone and the pastern) and hoof and cause lameness. If the animal is not immediately moved to an ergot-alkaloid free diet, loss of the affected tissue will occur. Looking at heifers consuming endophyte-infected tall fescue, Jacobson *et al.* [52] reported a “blood vessel congestion” and perivascular hemorrhage leading to vasoconstriction and gangrene of the tail. This was later associated with a decrease in temperature and blood flow to the tail [53]. Calves that received an ergot alkaloid containing crude extract made from tall fescue hay developed lameness and swelling and reddening of the coronary bands of the hoof and discoloration of the tail [45]. These observations were related to microscopic images of associated blood vessels that had thickened walls and a reduced lumen area. There is *in vitro* evidence that ergot alkaloids can be attributed to excessive smooth muscle growth leading to the observed thickening of the blood vessels [54]. More recently, studies using Doppler ultrasound have demonstrated that exposure to ergot alkaloids reduces the caudal artery luminal area in the tail of cattle [55], and the auricular artery luminal area in goats [56]. Thus, the cause of gangrenous ergotism seems to be a perturbation in the regulatory mechanisms that control or regulate blood flow to the affected extremities.

### 3.2. Pathogenesis

The looming question that remains is what triggers this acute response? This clinical form of ergotism has not been heavily researched in recent years due to the inconsistent nature of its occurrence. Most studies are halted upon initial observation of these symptoms on animal welfare grounds. Additionally, there is no evidence that helps explain why only subsets of a population exposed to ergot alkaloids develop this gangrenous form of ergotism. For example, a clinical report documenting a purported instance of fescue foot in buffalo cows reported that, out of a herd of 90 cows, three developed swelling lameness in the hindlimb [57]. In general, it is thought that the quantity of alkaloid considered to be toxic is closely related to environmental temperatures and development of gangrenous ergotism is exacerbated in colder climates [46]. As blood flow is reduced as a result of ergot alkaloid-induced vasoconstriction, the inability of the animal to thermoregulate intensifies the symptoms of hypothermia in cold weather. Additionally, as nitrogen-based compounds, increased production of ergot alkaloids has been associated with N fertilization of pastures. This will be discussed further in the fat necrosis section of this review. Thus, management practices like this and the interaction with large changes in local environment (e.g., sudden changes from periods of drought to excessive precipitation, or large shifts in temperature) should also be considered when assessing the cause of gangrenous ergotism in livestock [58].

## 4. Decreased Animal Productivity

The symptomology of decreases in livestock production associated with ergot alkaloid exposure have been well documented in previous reviews [1,2,59,60,61,62,63]. While the above gangrenous ergotism or fescue foot is a likely consequence of an acute exposure to ergot alkaloids, the gradual onset of decreased livestock productivity is a result of chronic alkaloid exposure.

### 4.1. Hyperthermia

The effects of heat stress have been difficult to separate from the effects ergot alkaloids have on livestock. This particular effect of ergot alkaloids may be more related to climate than other effects. Many of the signs of ergot alkaloid exposure overlap with signs of heat stress (depressed feed intake, elevated rectal temperatures, panting, elevated respiration rates). This is a large challenge when attempting to separate or study the interaction of heat stress to ergot alkaloid intake, as they both affect commonly measured variables and can confound one another. Regardless, animals consuming ergot alkaloids have a reduced capacity to maintain a thermoneutral body temperature that is exacerbated by sudden changes in the environmental temperatures [64]. Specifically, exposure to ergot alkaloids results in a decrease in the diurnal variation of core temperature, as shown in cattle receiving 0.021 mg of ergovaline/kg BW^0.75^ under heat stress conditions (31 °C) [65]. This results in a reduced tolerance by the animal for temperatures out of their narrowed thermoneutral range. Steers consuming ergot alkaloids have been shown to have a reduced ability to remove body heat through surface evaporation [66]. Also, the general effect of vasoconstriction caused by ergot alkaloids contributes to this by limiting the diffusion of heat via restricting blood flow to the periphery and the surface of the skin [67]. Thus, ergot alkaloids affect the cardiovascular system separately from the influence of environmental temperature and have been shown to worsen the consequences of heat stress [68]. Although hyperthermia itself is not a productive loss, it does play a role in the overall effect ergot alkaloids have on livestock (e.g., exacerbates decreased intake and reproduction) and certainly contributes to decrease productivity.

### 4.2. Decreased Weight Gain and Intake

For most livestock industries, liveweight gain is of primary economic importance. Cattle have gained from 30% to 100% less on ergot alkaloid-containing endophyte-infected tall fescue compared to cattle consuming an endophyte-free tall fescue diet [60]. In terms of weight gain, reduced animal performance on endophyte-infected tall fescue pastures and the corresponding consumption of ergot alkaloids while grazing is linked to a decrease in nutrient or dry matter intake [66,69,70]. Nutritive values between endophyte-infected and endophyte-free tall fescues are similar, implicating ergot alkaloids as causative agents [71]. Generally for ruminants, when feed intake decreases then apparent digestibility should increase [72]. Studies that have compared digestibilities of ergot alkaloid containing and ergot alkaloid free diets have yielded mixed results with increased [73,74,75] as well as decreased [66,70,76] digestibilities (Table 2). There are numerous possibilities that could explain this difference in outcomes that range from quality of hay or seed used, ergot alkaloid (measured primarily as ergovaline) intake concentrations, different environmental temperatures, ruminal flow kinetics, or *ad libitum versus* fixed intake levels. Studies that fixed dry matter intake across ergot alkaloid-containing and ergot alkaloid-free diets reported lower dry matter [66,70,76] and crude protein [70,76] digestibilities. Depressed digestibility is a function of the competition between rate of passage and digestion of a feedstuff [77]. In terms of passage, reductions in liquid passage and a tendency for reduced particulate passage rates have been reported in steers receiving ergovaline [75]. Also, as ergovaline increases in the diet particulate, outflow is reported to decrease [73,78]. These results have led to the conclusion that cattle consuming ergot alkaloids such as ergovaline or ergotamine gain less because they eat less [75].

**Table 2:** A summary of ergot alkaloid concentrations, the intake levels reported on a metabolic body weight (BW^0.75^), and the associated effects related to intake and digestibility.

Ergovaline Intake (mg/kg BW^0.75^) ^1^	Concentration (mg/kg DM)	Animal	Effect	Source
0.008 ^2^	0.120 ^3^	Steers	Decreased dry matter intake and digestibility, no difference in NDF digestibility	[70]
0.021	0.285	Steer calves	Decreased dry matter intake and digestibility, no difference in NDF digestibility, increased water intake	[66]
0.044	0.475	Steers	Intake equal, decrease in rumen fill observed with alkaloid, passage rate not affected	[78]
0.051	4.1	Steers	Intake was fixed, increase rumen fill observed (% DM)	[79]
0.053	1.17	Wethers	Decreased dry matter intake and digestibility, altered rumen fluid kinetics	[80]
0.057	0.96	Ram lambs	Equal intake, lower rumen fill	[81]
0.059	4.45	Steers	Intake was fixed, increase in rumen fill (% DM) observed and ruminal VFA concentrations, decreased blood flow to rumen	[82]
0.098	NA^4^	Steers	Intake was fixed, increased rumen fill observed (% DM), decreased particulate passage	[75]
0.19	3.0	Wethers	Decreased NDF digestibility, decrease in rumen fluid volume, increased water intake	[83]
0.093 ^2^	0.620 ^5^	Cows	Higher quantities of undegraded protein and NDF digestibility measured at the duodenum, increased ruminal VFA and ammonia nitrogen concentrations	[74]

^1^ All referenced levels are for ergovaline intake except for the reference below the dashed line that is for total ergot alkaloid intake; ^2^ Estimated intake level; ^3^ As-fed concentration; ^4^ Not available (NA); ^5^ Ergot-contaminated rye fed that contained ergonovine, ergotamine, ergocornine, ergocryptine, ergocristine, and ergosine.

If animals gain less because they eat less, then what is driving the decline in intake? Recent studies using pair-fed animals to eliminate difference in intake have reported increases in both rumen dry matter content and percent dry matter in ergot alkaloid treated cattle [75,79,82]. This may be a consequence of the reduced particulate passage and contributing factor in the depression of feed intake associated with ergot alkaloid exposure. Additionally, several studies have demonstrated elevated ruminal VFA concentrations in animals that have been exposed to an ergot alkaloid treatment [74,75,78,82]. A decrease in blood flow to the rumen epithelium and a corresponding decrease in acetate flux across this tissue were both associated with the presence of ergovaline and is a likely explanation for elevated VFA levels [82]. An additional explanation for these observations is again the reductions in particulate and liquid passage rates (for the increased DM and VFA concentrations, respectively) in the gastrointestinal tract. There are several *in vitro* studies that support this idea. An increased contractile tension of isolated rat colon has been observed when both ergotamine and lolitrem B were applied together, which suggested a synergistic interaction that could alter motility *in vivo* [84]. Intravenous administration of ergotamine or ergovaline has also resulted in an immediate inhibition of A and B contractions of the reticulum and rumen and increased the baseline tone of these compartments [85]. These observed effects could be due to direct actions of ergopeptine alkaloids like ergotamine and ergovaline on myenteric neurons and on smooth muscle [86]. Because ergot alkaloids are known to interact with serotonin receptors in vascular smooth muscle [31,33,87], and that serotonergic receptors are also involved in the regulation of gut motility [88,89,90], has led to speculation that ergopeptides could be interacting with serotonergic receptors of the gut, thereby negatively affecting motility and passage rate.

Because of the acute nature of the intravenous and parenteral administration of the ergot alkaloid treatments in the work by Poole *et al.* [86] and McLeay and Smith [85], Egert *et al.* [91] evaluated the effect of ergot alkaloids on foregut motility using a chronic dose of ground tall fescue seed applied daily via a rumen cannula using an indwelling remote telemetry pressure transducer. Ruminal administration of the ergot alkaloid treatment resulted in a more gradual and sustained alkaloid exposure to the animal. Perhaps as a result of this, the ruminal presence of ergot alkaloids did not have a significant effect on the various aspects of rumen motility using this system or treatment regime. An *in vitro* evaluation of gene expression in gastrointestinal smooth muscle using steers that were treated in the same manner as Egert *et al.* [91] reported lower levels of the serotonin receptor (5HTR) 5HTR2A and 5HTR4 RNA transcripts from steers exposed to an ergovaline containing diet when compared to controls [92]. Since both of these receptors are associated with contractility/motility of gut smooth muscle, the decrease in expression of the genes associated with these receptors can be interpreted as a decrease in motility caused by ergot alkaloid exposure. Much of this work, however is ongoing, and is designed to further the understandings of mechanisms responsible for reduction of intake and weight gain that result from ergot alkaloids fed to livestock.

### 4.3. Decreased Reproduction

The effect of ergot alkaloids reducing livestock reproductive performance has been reported and reviewed previously, with particular emphasis on the female gender [1,93]. Research has indicated that there is a broad disruption of the reproductive homeostasis in both sexes of various species of livestock. Like much of the other described impacts of ergot alkaloid exposure, the extent of this disruption has not been consistently observed. This is due to both direct and indirect effects of ergot alkaloid exposure through regional vasoconstriction and corresponding decreases in blood flow to reproductive tissues, decreases in dry matter intake, and/or increased body temperature.

#### 4.3.1. Lowered Prolactin

Decreases in serum prolactin have been associated with and used as an indicator of ergot alkaloid exposure in cattle [94], sheep [95], and horses [96]. The ergot alkaloid-induced suppression of prolactin in the anterior pituitary [97] is caused by the similarity of the ergoline ring portion of the ergot alkaloids to dopamine (Figure 1b) allow them to interact with D2 dopamine receptors [22]. This mimics the binding of dopamine and results in the observed decrease in prolactin. In light of many of the other symptoms of ergot alkaloid exposure being inconsistently expressed in livestock, this relatively consistent response of prolactin depression [62] has resulted in its use largely as an indicator of exposure. Given the diverse functional nature of the prolactin hormone, it is likely that this suppression of prolactin has an influence in other aspects of ergotism in livestock. Prolactin has been functionally linked to the initiation of lactation and mammogenesis [98]. Although decreased serum prolactin in lactating animals does not directly equate to decreased milk production [93], milk production declines have been associated with ergot alkaloid consumption in cattle [8,99] and sheep [100], and agalactia in horses [96]. Cattle that graze ergot alkaloid-containing tall fescue typically exhibit a shaggy hair coat even in the heat of summer, exacerbating heat stress associated with ergotism [101]. Elevation of prolactin associated with day length has been related to shedding in a number of species including horses [102]. Work in cattle grazing tall fescue indicated that prolactin concentrations were too low to initiate the shedding of the winter hair coat, likely delaying the onset of the summer hair coat growth [103]. Strickland *et al.* [62] suggested that the role of prolactin was much broader than merely as an indicator of disease. Strickland *et al.* [1] went on further to suggest that decreased prolactin levels are involved in the reduced reproduction of seasonal breeding animals more than nonseasonal breeding animals such as cattle. The role that lowered prolactin plays in terms of ergot alkaloid exposure is an area than requires continued research.

#### 4.3.2. Female-Specific Effects

In addition to a lowered prolactin level, female livestock have a lower level of progesterone in response to exposure to ergot alkaloids in tall fescue or in animals fed ergotamine [1]. This has been observed in heifers [104,105], cows [106,107], ewes [108] and mares [96]. Given the importance of progesterone in the establishment and maintenance of pregnancy, and the importance of producing live young to the financial success of most livestock operations, the effect of ergot alkaloids on the endocrinology of pregnancy is an aspect that has been evaluated from conception to birth. Cows that were exposed to ergot alkaloids via tall fescue grazing had a 41% lower conception rate than those grazing an ergot alkaloid-free pasture [109]. Waller [63] reviewed the effects of tall fescue alkaloids on pregnancy rates in cattle with an emphasis on the time in the reproductive cycle when ergot alkaloids have the greatest impact. In beef cattle, the period of concern for ergot alkaloids negatively affecting conception was identified as the time between ovulation and the first six days of embryonic development [105,107,110].

Research into the actual mechanisms behind the lowered progesterone is limited. Strickland *et al.* [1] speculated that the reported reductions in progesterone (a cholesterol based hormone) could be a result of lowered serum cholesterol observed in steers following consumption of ergot alkaloids in endophyte-infected tall fescue [111]. Another proposed explanation has been a reduction in blood flow. As detailed earlier in this review, ergot alkaloids interact with various receptors throughout the body, which can cause localized vasoconstriction. There has been speculation that a decrease in ovarian or luteal blood flow inhibiting the distribution of progesterone to systemic circulation might be an explanation for the absence of a difference in progesterone concentrations from luteal extracts collected from ergot alkaloid-containing or ergot alkaloid-freed diets [104]. Research directly confirming either of these concepts has yet to be reported in any species of livestock.

Research documenting the effect that ergot alkaloids have on the gonadotropins is limited and somewhat variable. However, given that luteinizing hormone (LH) and follicle stimulating hormone (FSH) are released from the anterior pituitary where an ergot alkaloid effect on prolactin secretion has been clearly demonstrated [97,112], an effect on gonadotropins is certainly possible. There are mixed reports on the effect that ergot alkaloids have had on LH. Subcutaneous doses of ergocornine administered to ewes did not have an effect on LH concentrations [113]. Conversely, a lowered LH release following exposure of cattle to ergonovine and ergotamine has been demonstrated [114,115]. Hodson *et al.* [116] evaluated the effect of bromocriptine (a dopaminergic receptor agonist derived from ergocryptine) on the LH and FSH secretory responses to gonadotropin-releasing hormone (GnRH) using ovine pituitary cell cultures. For both hormones, bromocriptine inhibited secretory response of the cells to GnRH. It was also demonstrated that this suppression of secretory response is accompanied by changes in the transcript levels of protein kinase C (PKC) and phospholipase C (PLC) two intracellular enzymes involved in the signaling cascades associated with 7-transmembrane bound G protein-coupled receptors such as dopamine receptors). This influence of ergot alkaloids on the intracellular signaling has been a hypothesized mechanism for many symptoms attributed to ergot alkaloids [117] and is a developing area of research that has implications including and reaching beyond the effects of reproduction.

Although ergot alkaloids have an impact on conception rates and disrupt normal endocrine function, what about their effects on the fetus in the remaining months of gestation? Fetal growth, organ weights, muscle weights and birth weights have all been shown to be lower in sheep as a result of maternal exposure to ergot alkaloids [118]. Reduced birth weight, associated with maternal ergot alkaloid consumption during gestation, has also been reported in cattle [119]. The vasoactivity of ergot alkaloids in bovine uterine and umbilical arteries would suggest that blood supply during gestation could be reduced due to vasoconstriction [31]. This would in turn limit nutrient supply to the fetus, resulting in retarded growth rates observed during gestation. These negative effects could persist throughout the offspring’s postnatal life given the impact of developmental programming during pregnancy related to significant amounts of variation for postnatal performance measures such as feed intake and carcass weights [120].

In contrast to cattle and sheep, on which significant impacts occur early in gestation, exposure of mares to ergot alkaloids during pregnancy results in prolonged gestation and late term foal losses, increased mare loss due to dystocia, and thickened placentas [121]. Gestation in mares consuming ergot alkaloid-containing tall fescue is reportedly extended by three to four weeks [96,122]. However, the foals carried past term exhibit signs of being premature and typically have poor muscling [93]. The frame size of these foals, in combination with a frequently incorrect presentation at parturition, results in a large percentage of cases of dystocia [122]. Severe cases of dystocia can result in the loss of the mare. The aforementioned thickened placentas (red bags) result from a prematurely separated placenta that is so named because the thickened placenta does not rupture during parturition, resulting in a blood-filled red color [50]. Foals are encased by this placenta and often asphyxiate without extensive human intervention [121]. If the mare and the foal survive gestation and parturition, then the lack of mammary development that is also associated with mares exposed to ergot alkaloids during gestation further limits the productivity (survival) of the foal. The absence of a placental lactogen in horses requires them to be essentially reliant on prolactin to stimulate prepartum lactogenesis [123]. Decreased milk production has been associated with consumption of ergot alkaloids [8,99], but ergot alkaloids have little observed effect on the placental lactogen in cattle or sheep. Consequently, the decreased prolactin combined with no placental lactogen cause mares to exhibit a high rate of agalactia (a complete absence of lactation) [96]. This will result in an incomplete transfer of passive immunity due to an insufficient supply of colostrum leaving the foal with an increased susceptibility to disease [121]. The unique reproductive impact on horses as compared to ruminant livestock may be a result in horses of the lack of pre-gastric fermentation and potential for detoxification of ergot alkaloids. This has the potential to result in differences in the population of intact alkaloids available for absorption, the concentration of alkaloids available for absorption, or site of absorption of ergot alkaloids in horses.

#### 4.3.3. Male-Specific Effects

Research into the reproductive effects of ergot alkaloids in male livestock has not been as well documented as for female livestock. There are studies, however, that demonstrate that male reproductive productivity may be altered as a result of ergot alkaloid exposure. Bulls fed ergotamine produced semen that appeared to have reduced fertilization potential [124]. Work from the same laboratory also demonstrated that semen collected from bulls grazing ergot alkaloid-containing tall fescue pastures used in *in vitro* fertilization resulted in embryos that had reduced cleavage rates, but mobility and morphology of the sperm were not affected at the times of collection and *in vitro* fertilization [125]. Conversely, a decrease in sperm concentration and increase in abnormal sperm collected from bulls grazing ergot alkaloid-containing tall fescue has also been reported [126]. Interestingly, sperm motility in semen samples collected from ergot alkaloid-exposed bulls after thawing has been reported to decrease, indicating that the ability of sperm cells to endure storage by freezing was negatively affected [126]. This negative effect on sperm motility has also been demonstrated with both ergotamine and dihydroergotamine [127]. In addition to reduced motility, sperm collected from bulls consuming ergot alkaloid-containing pasture also had a slower velocity [128]. The presence of the prolactin receptor in the testes and the presence of prolactin in seminal fluid has been demonstrated in cattle [129]. Given the effects of ergot alkaloids on prolactin in the pituitary, this may be an area where ergot alkaloids could have an effect on male reproduction. Another aspect worth considering is the localized effect of vasoconstriction on the temperature of the testes, as bulls consuming ergot alkaloids have a reduced scrotal temperature [125]. The effect ergot alkaloids have on the micro-RNA expression in bovine sperm has been investigated, but only slight alterations have been reported [130]. Regardless, this continues to be an area of interest in terms of male fertility as well as understanding the role of micro-RNAs in fertilization, initial embryonic development, and the ontogeny of ergot alkaloid exposure.

## 5. Fat Necrosis

In terms of the effects that ergot alkaloids have on livestock productivity, fat necrosis (abdominal lipomatosis) is the least studied and perhaps the most underreported. It has, however, been shown to occur in cattle [131], goats [132], deer [133], pigs [134], and horses [135]. Fat necrosis is defined by the occurrence of hard masses of necrotic fat in the mesentery of the intestinal tract that occupies a limited space in the abdomen and can interfere with normal passage of digesta, reproductive capacities, and parturition [61,63]. Consequently, the presence of fat necrosis may go undiagnosed, as decreases in intake and reproductive complications have been more associated with the reduced productivity associated with ergot alkaloids and not this specific aspect. The pathology of fat necrosis has been associated in cattle with grazing ergot alkaloid-containing pastures that have received a high rate of *N*-fertilization (≥700 kg N/ha/year) [136]. This was largely associated with the practice in the U.S. of fertilizing tall fescue pasture with poultry litter. Clinical cases seemed to subside with the cessation of this practice. The occurrence of these masses of necrotic fat has been shown in adult Eld’s deer (*Panolia eldii*) that appeared to coincide with the constriction of the ureters in some of the necropsies that resulted in renal complications [133]. A report of abdominal fat necrosis in a Pygmy goat (*Capra aegagrus hircus*) that had been on an ergot alkaloid-containing pasture observed compression of the rumen, small intestine, spiral colon, and gall bladder [132]. Outward symptoms prior to euthanasia were lethargy, anorexia and abdominal distension. A histologic examination of the masses revealed that in addition to adipocyte necrosis, infiltration of macrophages and lymphocytes had also occurred [132]. These studies [132,133,136] are all post-mortem clinical cases, but it is possible that, even if fat necrosis is not directly observed, adipocytes are being altered by exposure to ergot alkaloids.

The accumulation of ergot alkaloids in subcutaneous adipose tissue has been proposed [137] and this occurrence could disrupt or alter lipid metabolism. Why does fat necrosis not occur at this adipose site? Necrotic fat lesions have only been found in the abdominal fat and not in the subcutaneous or pericardial fat depots [17]. Cattle consuming an ergot alkaloid-containing fescue were more sensitive to an endotoxin challenge, indicating that an increased inflammatory response could be associated with ergot alkaloid consumption [138]. Given the enhanced inflammatory responses observed in the necrotic lesions detected in the above Pygmy goats [132] as well as in the abomasum, small, and large intestine of sheep orally dosed (1 mg/kg BW) with ergotamine [139], one possibility is an increased intestinal permeability to microflora. The presence of ergovaline did not have an effect on bovine rumen epithelial barrier function [140]; however, this work has yet to be repeated using the small intestinal epithelium. The effects of other mycotoxins have on modulating intestinal function has been evaluated and shown that other mycotoxins like deoxynivalenol (DON) are capable of increasing intestinal permeability [141]. This could permit translocation of bacteria normally restricted to the lumen of the gut that could cause the localized lesions associated with fat necrosis. However, more work is needed on this understudied effect of ergot alkaloid ingestion in livestock to determine if it contributes to chronic production losses.

## 6. Conclusions

There is not a single toxin that is solely responsible for the impact of ergot alkaloids on livestock. Rather, it is the collective impact of ergot alkaloids derived from external spore producing fungi (*Claviceps* spp.) and the endophytic fungi (*Epichloë*/*Neotyphodium* spp.) that are responsible for the broad spectrum of associated animal responses. Much of the available animal data addresses the symptoms and defines the problem. Presently, a significant pool of data addressing the cellular and biochemical impacts of ergot alkaloids on livestock is becoming available. A true solution to the complications associated with ergot alkaloid consumption will only become a reality when the work in the laboratory and the pasture/feed bunk converges.
